# Bio-Inspired Approaches—A Leverage for Robotics

**DOI:** 10.3390/biomimetics10070417

**Published:** 2025-06-27

**Authors:** Swaminath Venkateswaran, Damien Chablat, Poramate Manoonpong, Julien R. Serres

**Affiliations:** 1 De Vinci Research Center, De Vinci Higher Education, 92616 Paris, France; 2 Research Center for Industrial Robots Simulation and Testing, Technical University of Cluj-Napoca, Memorandumului 28, 400114 Cluj-Napoca, Romania; 3 Nantes Université, CNRS, LS2N, UMR 6004, F-44000 Nantes, France; 4 Embodied AI and Neurorobotics Laboratory, SDU Biorobotics, The Mærsk Mc-Kinney Møller Institute, The University of Southern Denmark, 5230 Odense, Denmark; 5 Bio-Inspired Robotics and Neural Engineering Laboratory, School of Information Science and Technology, Vidyasirimedhi Institute of Science and Technology, Rayong 21210, Thailand; 6 Aix Marseille University, CNRS, ISM, 13284 Marseille, France; 7 Institut Universitaire de France, IUF, 75231 Paris, France

The field of bio-inspired approaches (also known as biomimetics or biomimicry) is a design approach whereby a product or process is inspired by elements of nature, such as plants or animals. Bio-inspired approaches serve as inspiration and motivation for many engineers and designers in their efforts to identify unexpected solutions to problems. These approaches have resulted in significant innovations in the aerospace, marine, and automotive industries. The domains of bio-inspiration and bio-mimetics have also been the focus of several studies in the domain of robotics. There are several examples of their use in the literature, including their implementation in snake-type robots for underwater inspection or in worm-type systems for industrial pipeline inspections. This Special Issue presents the recent advancements in the domain of bio-inspired robotics and their potential applications in industry. This will help researchers from all communities to understand the relevance of bio-inspiration in robotics and will serve as a platform for the application of these cutting-edge approaches in other fields. This Special Issue comprises 11 articles in the domain of biomimetics, examining the study of this concept and its application in robotic systems.

In paper [1], the authors exploit the locomotion of bio-primates, which include continuous brachiation and ricochetal brachiation. The design mimics the transverse movements of sports climbers holding onto horizontal wall ledges. Based on a two-hand release design, a novel transverse ricochetal brachiation mechanism exploits inertial energy storage to enhance moving distance. Experimental prototypes helped to predict the success of subsequent locomotion cycles.

In paper [2], the authors present an angle sensor based on step-index profile plastic optical fiber (SI-POF), which was cost-effective and durable. The performance of the POF sensor was evaluated by measuring sensitivity and resolution in order to verify its reliability under extreme conditions, especially underwater. This study is vital to the development of biomimetic robot industry where existing sensors are difficult to deploy.

Paper [3] presents a novel whisker-sensing disk designed for 3D mapping in unstructured environments. The modeling is performed based on analytical and data-driven approaches to predict rotation angles based on magnetic field measurements. Experiments were conducted to validate the modeling, and the results highlight the effectiveness of 3D mapping in complex environments for robotic platforms in the future.

Paper [4] presents a review of robot control in spaces with low material and structural stiffness, which is usually challenging. Based on an in-depth review of scientific articles, the conclusions of this study indicate three top performing methods. The methods are based on minimizing control effort usage, tracking error mean, and tracking error deviation with some improvements in performance measures.

In paper [5], the authors propose the use of impedance control for the regulation of dynamic response of pneumatic soft robots, an approach already in existence for rigid robots. A non-linear discrete sliding mode impedance controller is formulated to control soft pneumatic robots. The controller does not require the manual tuning of parameters and can automatically calculate them based on impedance value. Experiments showed that the proposed controller can effectively limit the amplitude of undesirable vibrations.

Paper [6] presents a distributed model predictive controller based on leader–follower approach for addressing the collaborative transportation control issue of dual humanoid robots. Network latency issue is a prominent problem due to unstable network conditions that affects the consistency of collaboration. A socket communication was constructed to resolve the latency issue. A distributed model predictive control helped consider cumulative errors that lead to the enhanced position tracking accuracy of dual-robot collaborative control.

In paper [7], the authors measured hand movements in stroke patients using MediaPipe and Fahrenheit to assess their criterion-related validity. Consistent results were observed in peak angle and velocity comparisons across severity stages. The study highlighted the importance of MediaPipe in paralysis estimation.

Paper [8] presents important research on malfunctioning joints that lead to a high degree of compactness, eventually resulting in benefits such as low mass, low moment of inertia, and low drag. It was found that this multifunctionality was achieved through various means, such as multiple degrees of freedom, multifunctioning parts, over-actuation, and reconfiguration. This research suggests that the agility of robots could be improved using multifunctioning to reduce the size and mass of robotic joints.

In paper [9], the authors present a motion prediction model developed using convolutional neural networks (CNNs) for the efficient identification of motion types at the initial states. A multi-axial robotic arm integrated with a motion identification platform was developed to interact with humans by emulating their movements. A control strategy for addressing non-linearities and cross-coupled dynamics of the robotic system was applied. The results show that the robotic arm was able to achieve adequate controlled outcomes, thereby validating the feasibility of such an interactive robotic system in effective bio-inspired motion emulation.

Paper [10] presents a novel bionic amphibious robot, AmphiFinbot-II. The robot’s swimming and walking components involved a compound drive mechanism that allows for the simultaneous control of the rotation of the track and the wave-like motion of the undulating fin. The performance was tested via different motion patterns through computational fluid dynamics simulations. Experiments were also conducted on land and underwater, and the results were consistent with the simulation. The findings showed that the proposed robot possesses excellent amphibious motion capabilities through a unified control approach.

Finally, paper [11] presents the empirical kinematic control and data-driven modeling of a soft swimming robot. The robot consists of six serial connected segments that can bend individually using segmental pneumatic artificial muscles. Experiments were conducted to gather position and velocity of spatially digitized points using Qualisys Tracking Manager 1.6.0.1. Through offline analysis, a new complex variable algorithm was proposed to extract a linear approximative model. The proposed algorithm helped in extracting linear and chaotic modes and aided in linearizing the overall system dynamics.

The guest editors would like to sincerely thank all the contributors who took a keen interest in our Special Issue. All the articles went through rigorous peer review to ensure a high-quality publication. We also thank all the reviewers for their valuable comments and revisions. In addition, we would like to thank the editors from MDPI for their support in the organization and publication of this Special Issue.

We believe that this Special Issue will help fellow researchers and industrial experts to gain an in-depth understanding of the importance of biomimetics and how it could be implemented for various applications.
